# Interactive Voice Response with Feedback Intervention in Outpatient Treatment of Substance Use Problems in Adolescents and Young Adults: A Randomized Controlled Trial

**DOI:** 10.1007/s12529-016-9625-0

**Published:** 2016-12-27

**Authors:** Claes Andersson, Agneta Öjehagen, Martin O Olsson, Louise Brådvik, Anders Håkansson

**Affiliations:** 1 Faculty of Health and Society, Department of Criminology, Malmö University, -205 06 Malmö, SE Sweden; 2 Faculty of Medicine, Department of Clinical Sciences Lund, Psychiatry, Lund University, Lund, Sweden

**Keywords:** Randomized controlled trial (RCT), Interactive voice response (IVR), Outpatient treatment, Adolescents, Young adults, Substance use disorder, Mental health problems

## Abstract

**Purpose:**

Substance use disorders and problematic substance use are common problems in adolescence and young adulthood. Brief personalized feedback has been suggested for treatment of alcohol and drug problems and poor mental health. This repeated measurement randomized controlled trial examines the effect of an interactive voice response (IVR) system for assessing stress, depression, anxiety and substance use.

**Methods:**

The IVR system was used twice weekly over 3 months after treatment initiation, with or without addition of a personalized feedback intervention on stress and mental health symptoms. Both IVR assessment only (control group) and IVR assessment including feedback (intervention group) were provided as an add-on to treatment-as-usual procedures (TAU) in outpatient treatment of substance use problems in adolescents and young adults (*N* = 73).

**Results:**

By using a mixed models approach, differences in change scores were analyzed over the three-month assessment period. Compared to the control group, the intervention group demonstrated significantly greater improvement in the Arnetz and Hasson stress score (AHSS, *p* = 0.019), the total Symptoms Checklist 8 score (SCL-8D, *p* = 0.037), the SCL-8D anxiety sub-score (*p* = 0.017), and on a summarized feedback score (*p* = 0.026), but not on the depression subscale. There were no differences in global substance use scores between the intervention group (feedback on mental health symptoms) and the control group.

**Conclusion:**

In conclusion, IVR may be useful for follow-up and repeated interventions as an add-on to regular treatment, and personalized feedback could potentially improve mental health in adolescents and young adults with problematic substance use.

## Introduction

Substance use disorders are common in adolescence and young adulthood, with lifetime prevalence rates around 8% for alcohol use disorders and 2–3% for illicit drug use disorders [1, 2]. Stress and mental health problems are associated with substance use [3–5], and there is a considerable overlap between substance use and mental health problems [6, 7], as well as between substance use disorders and mental health disorders [8–12]. Self-medication of psychiatric symptoms may be a common pathway to substance use disorders in adolescence [13].

Treatment retention is generally low among adolescents and young adults treated for substance use [14]. Co-occurring conditions increase severity and complicate recovery [15, 16], and this has resulted in recommendations for integrated treatment of substance use disorders and comorbid conditions [17]. Recent reviews [18–20] have shown that integrated treatment is a promising approach, although the limited number of trials, inconsistent results, and difficulties of integrating with regular substance use treatment, warrant further research.

Relapse prevention [21] is an effective cognitive-behavioral intervention approach recognizing the close relationship between affective states and substance use [22–24]. It is designed to help individuals limit substance use associated with high-risk situations, such as negative affective states, during and after substance use treatment. The intervention includes a continuing care approach where important elements are support of self-control strategies, including identification and awareness of early warning signals, and enhancement of self-efficacy through feedback on the individual’s performance [25, 26].

Brief interventions, in their shortest form, only include assessment and clear and direct feedback, helping individuals become more aware of risks and think differently about the problematic behavior. Nevertheless, such interventions have been used successfully to reduce substance use, improve mental health, and increase motivation [27–29]. Providing mental health and substance use patients with systematic and continuous feedback during treatment is known to result in a positive development over time [30, 31].

Computerized interventions have offered small but significant effect sizes on outcomes such as substance use, treatment retention and adverse events, as well as improvements in therapeutic alliance and engagement in treatment [32]. Interactive voice response (IVR) is a well-established technology where a central computer uses pre-programmed scripts to be used in interaction with the user via their own mobile telephone handset [33]. IVR could therefore be used as a brief cognitive intervention on co-occurring affective states during substance use treatment. In the field of substance use and misuse, IVR has primarily been used for follow-up of substance use [34–36], while only a few feasibility studies report actual intervention results [37–40] and, to the best of the authors’ knowledge, no such study includes clinical samples of adolescents and young adults.

IVR has several advantages over similar technology, such as text messaging and smartphone applications, which have also been used for assessments or considered for interventions in the field of substance use [41, 42]. The IVR calls are natural reminders that increase the probability of response. Collected data are immediately secured on the server and can be used for analysis and action. No information is stored on the handheld device when using IVR, which is especially important when handling sensitive information, such as the individual’s mental health and substance use.

Our research group has used IVR to study the relationship between alcohol consumption and daily stress, symptoms of depression and anxiety [43], and for brief automated alcohol interventions [44] in young adults. IVR has also been used to monitor clinical samples of patients with mental health problems in primary care [45], and adolescents discharged from inpatient and outpatient psychiatric treatment [46, 47].

In a recent study, we used IVR to give brief automated feedback to prevent relapse in paroled offenders as add-on to the regular supervision offered by the Prison and Probation Services [48]. The study used a repeated-measures design where IVR was used daily to monitor stress, mental health, and substance use in participants over 30 consecutive days following parole. The intervention group received immediate feedback on the trend of summary scores for stress, mental health and substance use variables, in conjunction with the daily IVR assessments. The data were analyzed using linear mixed models, and the intervention group showed greater improvement over time than the control group in the summary scores, in mental health symptoms, in alcohol drinking, in substance use, and in ratings of the most stressful everyday events.

The present study in adolescents and young adults initiating substance-use treatment is based on our experiences from our previous study in paroled offenders [48]. A repeated measurement randomized controlled design was used to investigate the effect of brief automated feedback on prevention targeting co-occurring negative symptoms as an add-on to substance use treatment. IVR assessments were made twice weekly over a three-month period, and the feedback intervention included stress and mental health variables to reduce mental health problems and possibly also substance use. In a previous report [49] only focusing on treatment retention, the feedback interventions did not significantly improve treatment retention; unplanned dropout was 24% in the intervention group compared to 14% in the control group (*P* = 0.374). No baseline data differed between dropouts and the others.

The aim of the present study was to investigate whether adolescents and young adults in substance use treatment, who were randomized to a brief automated feedback intervention over a three-month assessment period, would show reductions in symptoms of stress, depression, anxiety, and a total summary feedback score of these measures. Another aim was to investigate whether a possible reduction of stress and symptoms would have an impact on simple substance-use scores assessing intensity of alcohol and drug use. We hypothesized that the feedback intervention on mental health symptoms would directly improve these symptoms, and indirectly improve substance use outcome.

## Materials and Methods

### Clinical Setting

Maria Malmö is an outpatient facility for treatment of substance use disorders and problematic substance use in Malmö, Sweden. The target group is adolescents and young adults, and the facility is managed by the social services and the health care services. Staff from both the social services and from the local department of psychiatry collaborate on evaluation and treatment, in compliance with national policies of a divided treatment responsibility between these two organizations. The facility has an upper age limit of 25 years, but no formal lower age limit. Treatment is an individualized psychosocial treatment, including pharmacological treatment as needed during early and protracted withdrawal. Further medical assessment and treatment is available, as well as referrals to further treatment or follow-up as necessary.

### Design

The present study was a randomized controlled trial in adolescents and young adults initiating treatment at the facility. Patients included in the study entered a treatment-as-usual (TAU) procedure with an add-on IVR, and were randomized into two groups. One group received personalized feedback on symptoms of stress, depression, and anxiety as part of the IVR procedure (TAU + IVR assessment + IVR feedback, i.e. the intervention group), and the other received IVR assessment only (TAU + IVR assessment, i.e., the control group). IVR in both groups was a twice-weekly assessment over a 3-month period. In the end of each call, the intervention group received a brief automated feedback on the development of their individual reported levels of stress, depression, and anxiety symptoms. The study was approved by the Regional Ethics Committee at Lund University (file number 2012-217), and was registered at ClinicalTrials.gov (NCT01706380).

Participants were recruited between October 2012 and December 2013. Patients were offered participation by the counselor during the first or second visit to the clinic, during which baseline assessment was carried out as part of treatment-as-usual procedures. Staff did not offer participation to patients with severe psychiatric disorders, severe intellectual disability, and difficulties understanding the Swedish language, and subjects would also be excluded if they could not register a private cell-phone number. A cell-phone was chosen because it was considered more private than a landline phone. The number of subjects excluded was reported by study staff to be low, and none were excluded due to inability to register a cell-phone number.

Willing participants met with a research assistant to sign written consent (from parent or guardians for those under the age of 15), and for an automated telephone baseline assessment, including registration of cell-phone number for use in the trial. The randomization into two groups was based on a 1:1 random allocation sequence and a fixed-block size of ten to ensure balanced study arms. All participants received compensation of 100 SEK (around 12 USD) on entering the study and another 100 SEK (12 USD) on formal completion of the study after 12 weeks.

### Assessment

The baseline assessment consisted of data derived from the standard baseline assessment used at the facility. This was a semi-structured Swedish questionnaire used in clinical assessment of adolescents and young adults with substance use problems, the adolescent version of the DOK documentation system [50]. Variables included socio-demographic data, substance use history and psychiatric problems. For diagnostic purposes, subjects were interviewed using the Mini Neuropsychiatric Interview (MINI) [51].

The baseline assessment and automated telephone twice-weekly IVR monitoring involved a total of 19 items, and one supplementary item for those reporting drug uses. The first 15 items assessed stress and symptoms of poor mental health, including depression and anxiety, and were summarized into a total summary feedback score, with a Cronbach’s alpha coefficient ranging between 0.89 and 0.97 throughout the follow-up period. Stress was measured with the Arnetz and Hasson stress questionnaire (AHSS), which involves seven items measuring common indices of stress [52]. In this study, the Cronbach’s alpha coefficient ranged between 0.74 and 0.95, compared with 0.79 in the original study [52]. Anxiety and depression were measured with the Symptoms Checklist 8D (SCL-8D) [53]. The Cronbach’s alpha coefficient on the total scale ranged between 0.88 and 0.97 compared with 0.80 in the original study, and between 0.74 and 0.95 on both the anxiety and depression subscales. All feedback items, i.e. AHSS and SCL-8D, were scored on a 0 (bad) to 9 (good) digit scale, so that increased scores indicated improved mental health.

In addition to the feedback items, a global measure was assessed to monitor the trajectory of alcohol and drug use during the assessment period. Four items measured any alcohol use and any drug use on the present and preceding day, where responses were given either as “no” (0) or “yes” (1). These four substance use variables were summarized into one alcohol use sub-score and one drug use sub-score (with each sub-score ranging from 0 to (2), and finally into one summarized substance score (ranging from 0 to 4). For all substance use variables, reduced scores indicate fewer days of substance use.

Finally, participants who had reported any drug use either on the present or preceding day were asked to record a message specifying the type of drug used. This question was not used in the present analyses, due to a low response rate.

### Monitoring

The IVR system attempted to monitor participants in both groups automatically, twice weekly over 12 weeks, thereby giving potentially 24 follow-up assessments plus the baseline assessment. Monitoring continued for 12 weeks regardless of whether participants continued treatment or treatment had been discontinued. Attempts were made to monitor participants every second hour between noon and 8 pm on Wednesdays and Thursdays (first weekly assessment), and on Saturdays and Sundays (second weekly assessment). The system was programmed to accept incoming calls from the participants on the same days, so that assessments could also be initiated directly by the participants. In both cases, i.e., both for outgoing and incoming calls, the participant had to confirm identification by entering their personal 10-digit identification number before hearing any information that could be connected to the research project, or before responding to any questions. When either of the two weekly assessments had been completed, no further attempts were made until the next scheduled weekly assessment. A text message (SMS) reminder was sent on Thursdays and Sundays at 11 am if the participants had not completed the scheduled assessment by that time. The SMS was brief and did not include information about the research project (“Hi, it’s Maria M. Wonder how you are? Please call XXX-XX XX XX”).

### Intervention

The intervention was inspired by relapse prevention and applied a brief intervention methodology. Participants in the intervention group received automated telephone feedback immediately after each follow-up assessment [48]. The feedback intervention targeted awareness of stress and mental health as potential triggers for substance use. It was based on a simple calculation of the total summary score for the current assessment, including the 15 items on stress and mental health symptoms (see above), compared with the total score for the same 15 items on the previous assessment. The respondent was informed about whether the current result was positive, negative, or neutral, after which the respondent was asked whether they perceived their development as positive, negative, or neutral. In cases where the calculation and/or the respondent indicated a negative direction, the respondent was recommended to talk with someone they trusted, for example their counselor at the treatment facility.

### Statistics

Descriptive statistics are presented as mean values and associated standard deviations (SD) for continuous variables, and as frequencies (percentage) for categorical data. Baseline characteristics of the participating subjects in the intervention group and the control group were compared using the Mann-Whitney test for continuous or ordinal variables and Pearson’s chi-squared test (*χ*2) for categorical variables. The study applied a repeated measurement design and the outcome measurements were the change in scores over a three-month period (24 assessments) on the following variables: AHSS, SCL-8D total score and sub-scores for anxiety and depression, respectively, the summary (AHSS and SCL-8D) score, alcohol and drug use scores, respectively, and the summarized substance use score. The outcome variables were analyzed using a mixed models approach considering repeated measures, and where group (intervention vs. control), time (assessment 1–24), age (below vs. above 18 years), and gender (male vs. female) were entered as fixed effects, and subjects as random effect (intercept) in the final model. In separate models, time x group, MINI substance use diagnosis (present vs. absent), and MINI non-substance use diagnosis (present vs. absent) were entered as fixed effects. For repeated measurements, an autoregressive covariance structure component (related to the individual’s repeatedly occurring responses) was pre-specified. To minimize the missing data, missing values from one or more items, including specific factor-representing scores, were imputed using the mean value of the remaining, non-missing, items. All statistical calculations were performed using SPSS statistical software (IBM SPSS Statistics for Windows, Version 22.0. Armonk, NY: IBM Corp).

## Results

### Participants

**Fig. 1:**
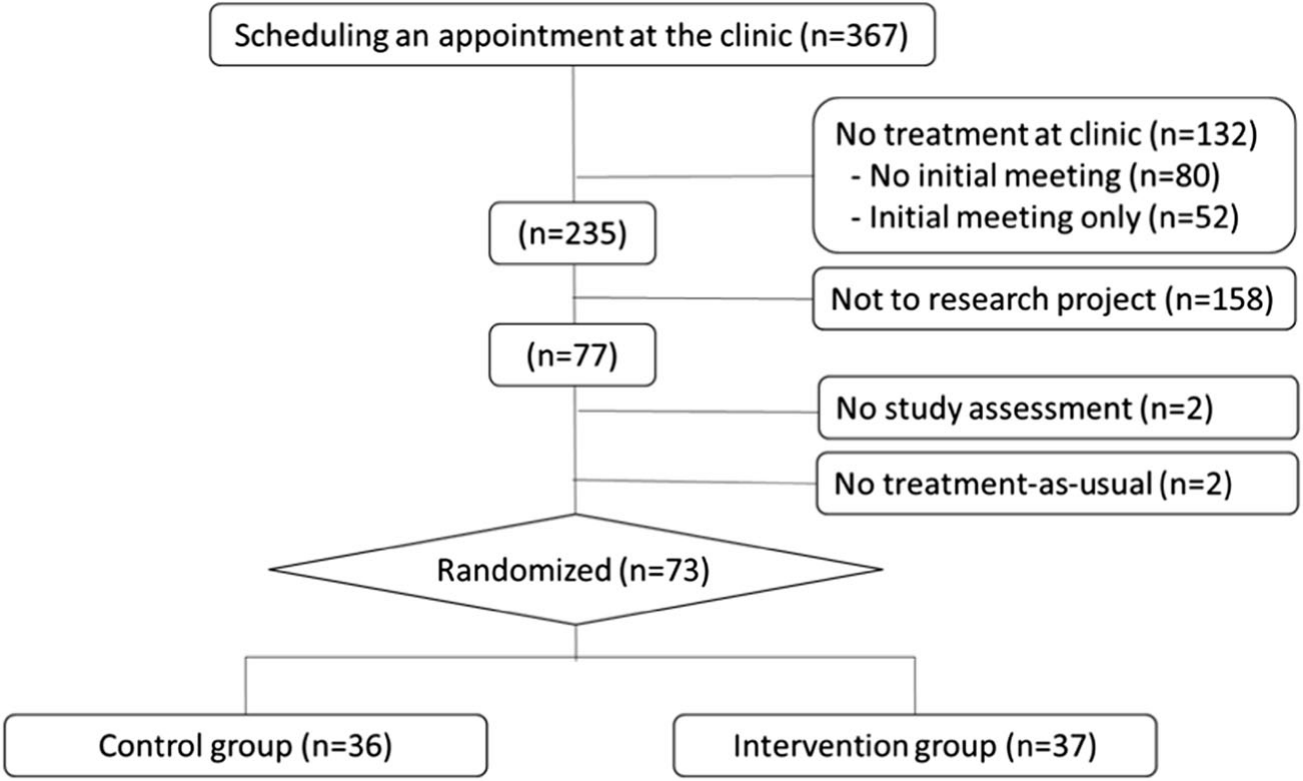
Flow-chart

As shown in Fig. 1, 367 subjects (30% women) were referred to and received an initial appointment at the facility during the recruitment period. Eighty patients (21%) did not turn up for their initial appointment, and 52 (36%) chose to discontinue treatment after one meeting and were not informed about the study. The remaining 235 were potentially eligible for participation in the study; of these, 158 did not participate, either because they were never formally approached with an offer to participate, or because they actively declined participation in the study. Seventy-seven subjects agreed to participate, but two subjects did not turn up for study assessments and another two did not initiate the regular treatment at the facility.

The final number of subjects was 73 (20% of all potential subjects), and these were randomized into the two groups, 36 into the control group (TAU + IVR assessment), and 37 into the intervention group (TAU + IVR assessment + IVR feedback). During the 12 weeks assessment period, there was no difference in number of days in treatment at the clinic between the control group and the intervention group (76.0 ± 17.5 vs 70.4 ± 23.6, *p* = 0.377). The representativity of our sample was analyzed by comparing baseline data [38] collected at the facility as part of the standard procedure with data concerning subjects not included in the study. These comparisons did not identify significant differences in terms of gender or the type of facility from which they were referred. Participants were also compared with subjects applying for treatment throughout the subsequent year (2014), and no significant differences were found in terms of gender, criminal convictions, or primary drug of abuse.

### Baseline Data

The random allocation was successful as no significant baseline differences were found between the two groups. As shown in Table 1, there were no baseline differences in gender distribution and mean age between the control group and the intervention group. There were no significant differences between groups with respect to regular tobacco use, baseline MINI substance use diagnosis, or MINI non-substance use diagnosis.

**Table 1:** By intervention group (*n* = 73); baseline characteristics of included subjects

	Control group	Intervention group	*p* value
Number	36	37	
Male gender	20 (56%)	27 (73%)	0.147
Age	18.1 (2.9)	17.7 (2.3)	0.772
Tobacco use	32 (89%)	26 (72%)	0.135
Any MINI diagnosis	33 (92%)	31 (84%)	0.479
MINI substance use disorder	33 (92%)	29 (78%)	0.190
Alcohol	7 (19%)	8 (22%)	1.000
Other substances	31 (86%)	28 (76%)	0.374
MINI non-substance-use diagnosis	24 (67%)	22 (59%)	0.630
Current depression	9 (25%)	10 (27%)	0.844
Agoraphobia	10 (28%)	7 (19%)	0.371
Generalized anxiety disorder	10 (28%)	10 (27%)	0.943
Antisocial personality disorder	12 (33%)	8 (22%)	0.262

Table 2 presents baseline data for the variables that were used for the twice-weekly IVR assessments in both the control group and the intervention group, including the variables that were used for feedback in the intervention group. No baseline differences were found.

**Table 2:** By intervention group (*n* = 73); baseline values on the variables used for daily assessments (AHSS, SCL-8D, alcohol, and drug use), a summary feedback score, and a summary substance use score, the latter not used for feedback

	Control group	Intervention group	*p* value
AHSS	42.1 (10.9)	42.8 (10.1)	0.969
SCL-8D	45.0 (19.2)	47.7 (15.3)	0.643
Anxiety	22.6 (10.5)	23.4 (9.0)	0.908
Depression	22.4 (9.6)	24.3 (7.4)	0.476
Total summary feedback score	87.2 (28.9)	90.5 (23.6)	0.817
Alcohol use	0.1 (0.5)	0.1 (0.3)	0.787
Drug use	0.3 (0.6)	0.2 (0.5)	0.734
Summary substance use score	0.4 (0.8)	0.3 (0.6)	0.925

Note: *AHSS* Arnetz and Hasson stress questionnaire, *SCL-8D* eight-item version of the Symptoms Check List

### Response Rates

Out of 1825 possible telephone assessments, 1009 (55.3%) were completed, 55.1% in the control group and 55.5% in the intervention group (*p* = 0.888). The total number of observations was 513 in the intervention group and 496 in the control group. The number of missing responses for the eight specific scores investigated varied between 3.1 and 7.4% in the intervention group, and between 2.0 and 11.3% in the control group. In the intervention group, 27–49% had at least one item missing in any of the specific scores for poor mental health, and the corresponding figure in the control group was 28–44%. At each complete assessment, numeric responses could be given to a total of 19,171 questions, of which 18,735 (97.7%) were responded to, 97.7% in the control group and 97.8% in the intervention group (*p* = 0.791). On 829 (82%) occasions, assessments were initiated by the IVR system, while assessments were initiated by the respondent on the remaining 180 occasions. Mean duration of the calls was 2.52 (SD 1.01) minutes in the intervention group (TAU + IVR assessment + IVR feedback) and 2.21 (SD 0.57) minutes in the control group (TAU + IVR assessment).

### Intervention Effect

The results of the final linear mixed models are presented in Table 3. Differences in change score over a three-month period (24 assessments) are shown for the intervention group (TAU + IVR assessment + IVR feedback) and the control group (TAU + IVR assessment) for the outcome variables. Compared with the control group, the intervention group demonstrated significantly greater improvement in AHSS stress score (*p* = 0.019), in total SCL-8D score (*p* = 0.037), in the anxiety subscale (*p* = 0.017), and in the total summary feedback score (*p* = 0.026) over the study period. There was no difference in change between the two groups on the SCL-8D depression subscale included in the feedback. The global substance use scores, not included in feedback, showed an improvement, while there were no differences between intervention and control group. These results were not altered when controlling for a time x group factor, or when controlling for the presence of a substance use diagnosis or a psychiatric diagnosis other than substance use (data not shown).

**Table 3:** By intervention group (*n* = 73); Mixed model analysis of repeated measures

	Estimate	df	t	*p* value	95% CI
AHSS	4.49	64.84	2.41	0.019*	0.77, 8.21
SCL-8D	5.20	64.94	2.13	0.037*	0.32, 10.08
Anxiety	3.23	63.91	2.44	0.017*	0.59, 5.87
Depression	1.98	65.21	1.60	0.114	−0.49, 4.45
Total summary score	9.40	65.15	2.28	0.026*	1.16, 17.65
Alcohol use	−0.02	50.77	−0.24	0.809	−0.15, 0.12
Drug use	−0.07	56.18	−0.94	0.351	−0.23, 0.08
Total substance score	−0.08	57.24	−0.08	0.452	−0.28, 0.13

Results presenting the difference in change over a 3-month assessment period (12 assessments) between the intervention group (TAU + IVR assessment + IVR feedback) and the control group (TAU + IVR assessment) on the following outcome variables: stress (AHSS), mental health symptoms (SCL-8D), SCL-8D sub-scores for anxiety and depression, the summary total score (including AHSS and SCL-8D) used for feedback in the intervention group, and a summarized total substance use score (including alcohol and drug use), the latter not used for feedback

*AHSS* Arnetz and Hasson stress questionnaire, *SCL-8D* eight-item version of the Symptoms Check List

*p < 0.05

## Discussion

The main result is that subjects receiving personalized feedback on mental health had a significantly greater improvement in scores of stress and anxiety symptoms during the assessment period. This confirms previous positive results of continuous and systematic brief personalized interventions on mental health variables, both in studies on relapse prevention [22–24] and personalized feedback [27–31].

There was no difference in the effects on depressive symptoms between the intervention and control group, which is consistent with some previous studies. In a study of patients with treatment for comorbid substance use and mood and anxiety disorders, an effect was seen for symptoms of stress and a trend towards an effect on anxiety, while depression was not altered [54]. Also, a previous study on a personalized feedback intervention in depressed individuals indicated that a favorable effect on depressive mood may be more difficult to obtain and may evolve only slowly [55]. Consequently, in populations like the one studied here, it cannot be excluded that depressive symptoms follow a different course in substance use treatment than symptoms of anxiety.

The positive results for symptoms of stress and anxiety did not apply to substance use, which contradicts the hypothesis in the present study. Previous research has presented inconsistent results on the effects of interventions on co-occurring conditions [18–20]. Here, possible reasons for the negative result may be the limited extent of the intervention, a weak relationship between the studied variables, or that identified improvements were not sufficient to influence substance use. Furthermore, the intervention period of 3 months was short. The result might also depend on our global assessment of substance use, i.e., the fact that we only assessed use (yes or no) on the current and preceding day. The dichotomized questions about alcohol and drugs may have been too unspecific to capture changes in consumption, compared to the 10-digit scale used in the study in paroled offenders [48]. A significant difference between the two studies is that the feedback in the previous study included substance use variables. Inclusion of substance use in the present feedback might have resulted in reduced substance use, as in the study on paroled offenders [48]. It should be underlined that our intervention was given as an add-on to the regular substance use treatment given to both groups. Both groups had equal reductions in substance use, which is probably a result of the regular treatment.

In the field of substance use disorders, among several IVR studies [34–36], few studies have reported intervention results [37–40]. The present study and our previous study on paroled offenders [48] are the first studies to report positive intervention effects from a continuous-care, brief personalized feedback intervention delivered by IVR, in vulnerable populations with a high degree of substance use and mental health problems. The personalized IVR feedback method, inspired by relapse prevention, used in both our studies holds promise as a potential add-on in the treatment of mental health problems in populations with problematic substance use or criminal behavior. More research is needed to examine whether the potential effect of this technique can be generalized to other settings and populations suffering from mental health problems and substance use problems.

The vulnerability of the study population is demonstrated by the high percentage of participants who fulfilled criteria for at least one MINI psychiatric disorder (other than substance use disorders) at baseline. Sixty-three percent of the subjects in the present study met criteria for at least one DSM-IV diagnosis other than substance use disorders included in the MINI interview. This clinical picture is consistent with previous data. In a literature review, Armstrong and Costello [8] showed that 60% of adolescents with substance use problems in clinical samples had comorbid psychiatric diagnoses. In the Swedish setting, a study from a different center demonstrated that 81 to 90% of adolescents seeking treatment for substance use problems fulfilled criteria for another mental disorder [11]. Based on this comparison, the findings from the present dataset regarding psychiatric comorbidity are likely to be generalizable to other groups of young people with problematic substance use.

The main strengths of the study are the randomized controlled design and that it shows a positive therapeutic effect on the symptoms that are common and central to young populations with substance abuse/dependence. The repeated measures design and use of mixed models is also a strength, offering statistical power when analyzing how outcomes change over time when affected by the feedback intervention [56].

All participants in the present study had access to their own cell-phone. The frequent use of cell-phones means that both follow-up and interventions can easily be implemented in adolescents and young adults in substance use treatment. Adverse events from IVR contact were not measured systematically, but none were mentioned spontaneously in follow-up assessments for patients remaining throughout the 12-week study period. Also, given the low level of complexity of the present follow-up and assessment technique, it can be assumed that any adverse events are mild.

The present study also has some limitations. The first and most important is the high rate of attrition preceding the random assignment to a group; this may have affected the results. Only 73 out of 367 patients (20%) entering the facility could be included in the study. One factor that might have contributed to attrition is that final inclusion in the study was arranged at a separately scheduled meeting with a research assistant. However, no major differences could be identified when comparing those initiating contact with the treatment facility and those finally participating in the study, and the population seems to be representative to similar populations [8].

A second limitation concerns the measurements of substance use. While the self-reported scores in the present study did not demonstrate significant improvement in the intervention group, systematic data for an objective follow-up measurement of substance use were not available in the present study.

A third inherent limitation of an automated telephone method is the limited amount of information that can be obtained during each assessment. This method favors the use of brief symptom scales rather than diagnostic tools, so symptoms such as psychotic manifestations could not be assessed over time in this study. However, the feasibility of using the present method, along with the potentially favorable results displayed here, holds promise as a method for frequent follow-up of briefly measured symptom scores, including central components such as depressive symptoms, anxiety, and stress.

A fourth limitation concerns analysis of the twice-weekly assessments that contained missing data. We used imputation for these missing values. These imputed values gave us gains in terms of a larger sample size and statistical power, but also a loss in the quality of data since imputation cannot replace missing information. Since the items included in each score are strongly or reasonably correlated to each other, we believe that the gain may outweigh any disadvantages.

In conclusion, a personalized feedback added to IVR assessments was a useful intervention for stress and anxiety during treatment for substance use disorders and problematic substance use in adolescents and young adults. The addition of automated personalized feedback may be a promising tool in alleviating mental health symptoms during treatment, although more remains to be studied about its potential role in treating the actual substance use disorder. If the findings can be replicated in future work, implications for adolescents and young adults with substance use problems may be significant.
